# Regulation of *ytfK* by cAMP-CRP Contributes to SpoT-Dependent Accumulation of (p)ppGpp in Response to Carbon Starvation *YtfK* Responds to Glucose Exhaustion

**DOI:** 10.3389/fmicb.2021.775164

**Published:** 2021-11-04

**Authors:** Laura Meyer, Elsa Germain, Etienne Maisonneuve

**Affiliations:** Laboratoire de Chimie Bactérienne, Institut de Microbiologie de la Méditerranée, CNRS-Aix Marseille Univ (UMR7283), Marseille, France

**Keywords:** (p)ppGpp, stringent response, *ytfK*, cAMP, CRP, glucose starvation, *E. coli*

## Abstract

Guanosine penta- or tetraphosphate (known as (p)ppGpp) serves as second messenger to respond to nutrient downshift and other environmental stresses, a phenomenon called stringent response. Accumulation of (p)ppGpp promotes the coordinated inhibition of macromolecule synthesis, as well as the activation of stress response pathways to cope and adapt to harmful conditions. In *Escherichia coli*, the (p)ppGpp level is tightly regulated by two enzymes, the (p)ppGpp synthetase RelA and the bifunctional synthetase/hydrolase SpoT. We recently identified the small protein YtfK as a key regulator of SpoT-mediated activation of stringent response in *E. coli*. Here, we further characterized the regulation of *ytfK*. We observed that *ytfK* is subjected to catabolite repression and is positively regulated by the cyclic AMP (cAMP)-cAMP receptor protein (CRP) complex. Importantly, YtfK contributes to SpoT-dependent accumulation of (p)ppGpp and cell survival in response to glucose starvation. Therefore, regulation of *ytfK* by the cAMP-CRP appears important to adjust (p)ppGpp level and coordinate cellular metabolism in response to glucose availability.

## Introduction

Bacteria have evolved efficient stress response mechanisms to quickly adjust cell growth and metabolism according to challenging environments. One of such bacterial responses is the near-universal stringent response. The hyperphosphorylated derivatives of GDP and GTP, guanosine tetra- and pentaphosphate (collectively named (p)ppGpp), are the central signaling molecules of the stringent response (Cashel and Gallant, 1969; Potrykus and Cashel, 2008). These alarmones allow rapid and robust stress adaptation by affecting gene expression and metabolism (Cashel, 1969; Potrykus and Cashel, 2008; Hauryliuk et al., 2015; Liu et al., 2015; Steinchen and Bange, 2016). Since, (p)ppGpp has also emerged as an important regulator of bacterial virulence, survival during host invasion (reviewed in Hauryliuk et al., 2015; Irving et al., 2021) and antibiotic resistance and tolerance (Nguyen et al., 2011; Amato et al., 2013; Helaine et al., 2014; Amato and Brynildsen, 2015).

The RelA–SpoT Homologue (RSH) family of bifunctional proteins is key players in synthesizing and degrading (p)ppGpp (Atkinson et al., 2011). Therefore, the tight balance between both reciprocal activities constitutes a crucial point of regulation for fine tuning (p)ppGpp homeostasis. The long RSH proteins share a similar domain architecture and can be divided into two regions of similar size. The N-terminal half of the protein harbors the catalytic synthetase and the hydrolase domains. The C-terminal half of the protein contains four regulatory (TGS, helical, CC and ACT) domains with essential role in sensing and transducing stress signal to the catalytic domains (Hogg et al., 2004; Hauryliuk et al., 2015; Pausch et al., 2020; Tamman et al., 2020). In most gamma and beta-proteobacteria, to which *Escherichia coli* belongs, the stringent response is driven by two paralogous RSH enzymes named RelA and SpoT. While SpoT has both functional synthetase and hydrolase domains, RelA is a monofunctional synthetase with a degenerated inactive hydrolase domain, making SpoT the primary source of (p)ppGpp hydrolysis (Xiao et al., 1991).

The (p)ppGpp synthetase activity of RelA is triggered in response to amino acid starvation *via* a ribosomal mechanism. Under this condition, deacylated tRNAs accumulate and activation occurs when RelA binds with an uncharged tRNA at an empty A-site of a stalled ribosome (Cashel and Gallant, 1969; Haseltine and Block, 1973; Arenz et al., 2016; Winther et al., 2018).

SpoT functions as a central protein which integrates various stress signals (Hernandez and Bremer, 1991; Xiao et al., 1991), other than amino acid starvation, such as fatty acid (Seyfzadeh et al., 1993), carbon (Xiao et al., 1991), iron (Vinella et al., 2005) and phosphate (Spira et al., 1995) starvations. Importantly, the hydrolysis activity of SpoT is crucial for balancing the basal activity of RelA. Indeed, disruption of the *spoT* gene in the presence of *relA* leads to a lethal accumulation of (p)ppGpp (Xiao et al., 1991; Baba et al., 2006). Therefore, a fine regulation of reciprocal SpoT activities is essential to correctly adjust intracellular (p)ppGpp level in response to bacterial surrounding.

Interaction of SpoT with other protein partners directly controls the balance between reciprocal activities. Indeed, it has been reported that the acyl carrier protein (ACP) binds the TGS domain of SpoT to promote (p)ppGpp accumulation during fatty acid starvation (Battesti and Bouveret, 2006). Interaction of SpoT with the CgtA/ObgE GTPase is proposed to modulate hydrolase activity during exponential growth (Wout et al., 2004; Jiang et al., 2007). In addition, SpoT hydrolase activity is promoted by binding to the anti-σ^70^ factor Rsd upon carbon downshift (Lee et al., 2018). Finally and more recently, we reported that the small protein YtfK can directly interact with the catalytic domains of SpoT to activate the stringent response under fatty acid or phosphate starvations by tilting the catalytic balance toward synthesis rather than hydrolysis (Germain et al., 2019). Moreover, the SpoT-YtfK ratio controls the switch of SpoT activities (Germain et al., 2019). Therefore, regulation of the level of YtfK protein is crucial for adjusting (p)ppGpp level in response to external stresses.

In this study, we dissected the *ytfK* promoter region and searched for new candidate genes involved in regulation of *ytfK* expression. Overexpression of one of these genes (*cpdA*), encoding a cAMP phosphodiesterase, strongly decreases *ytfK* expression. Importantly, our results show that the cAMP-CRP complex directly binds the *ytfK* promoter region to positively regulate its transcription in response to glucose availability. Moreover, we show that YtfK contributes to SpoT-dependent accumulation of (p)ppGpp and cell survival during glucose deprivation. Therefore, regulation of *ytfK* by the cAMP-CRP complex seems to play an important role in sensing and transducing signal to SpoT to coordinate cellular metabolism in response to glucose availability.

## Materials and Methods

### Bacterial Strains, Media, and Growth Conditions

Bacterial strains used in this study are listed in Supplementary Table S1. *E. coli* DH5α strain was the general cloning host. All *E. coli* strains were derived from MG1655 strain and grown at 37°C in LB (Lysogeny Broth) liquid medium from Oxoid (LP0021B and LP0042B; Clark and Maaløe, 1967) or NA (Nutrient Agar) solid medium from Oxoid (CM0003B). M9 minimal liquid medium was composed of M9 salt (60mM Na_2_HPO_4_, 22mM KH_2_PO_4_, 8mM NaCl, and 20mM NH_4_Cl), 1mM MgSO_4_, 100μM CaCl_2,_ 1μg/ml thiamine, and 0.025% or 0.2% glucose. MOPS minimal liquid medium was prepared as previously described (Neidhardt et al., 1974) and was free of nucleobases and amino acids. When necessary, media were supplemented with 80μg/ml X-gal (5-bromo-4-chloro-3-indolyl-β-D-galactopyranoside) and antibiotics used at the following concentrations: 50μg/ml ampicillin, 50μg/ml chloramphenicol, and 25μg/ml kanamycin. P1 transductions were performed as previously described (Thomason et al., 2007).

Expression of lambda recombinase from pKD46 (Datsenko and Wanner, 2000) was induced by adding 0.2% of arabinose and by growing cells at 30°C during 1.5h, pKD46 plasmid was then eliminated by streaking colonies on NA solid medium and by incubating plates overnight at 37°C. Kanamycin resistance cassette was flipped out as previously described (Cherepanov and Wackernagel, 1995).

### DNA Manipulations

Plasmids used in this work are listed in Supplementary Table S1 and were extracted using Monarch plasmid miniprep kit (Biolabs). PCRs were carried out from colonies with Phusion DNA polymerase (Thermo Scientific) to amplify DNA fragments used for cloning or strain constructions and Gotaq flexi DNA polymerase (Promega) for diagnostic PCR. PCR products were purified using the Monarch DNA gel extraction kit (Biolabs).

### Plasmid Constructions

The plasmid derivatives used in this study (Supplementary Table S1) were constructed by amplifying genes by PCR from template chromosomal DNA using primers listed in Supplementary Table S2 and by digesting DNA with restriction enzymes indicated in Supplementary Table S2.

### Construction of Reporter Strains

Transcriptional and translational fusions reporter strains were constructed by two-step λ red-mediated recombination, adapted from Blank et al. (2011). A chloramphenicol resistance cassette was amplified by PCR together with an I-*SceI* recognition site using pWRG100 plasmid as template with primers (357/358) for translational and (353/354 or 577/354) for transcriptional fusions (Supplementary Table S2) containing a 50bp homologous sequence with upstream and downstream of the *ytfK* locus. MG1655 strain harboring pKD46 plasmid was electroporated with the resulting fragment and the insertion of the I-*SceI*:*cat* fragment into the target locus was verified by PCR and then P1 transduced into the TB28 strain. The resulting strain was then transformed with pWRG99 harboring the I-*SceI* endonuclease under the control of an anhydrotetracycline inducible promoter. The chloramphenicol cassette was then removed by counter selection using lambda red recombination to insert a PCR product complementary to the flanking regions of the I-*SceI*:*cat* cassette on the chromosome. The PCR product of the different transcriptional fusions was generated with primers (355/356) and pGH254:P_*ytfK* P1+P2_:*lacZ* or pGH254:P_*ytfK* P2_:*lacZ* as templates or primers (708/356) and pGH254:P_*ytfK* P1_:*lacZ* as template (Supplementary Tables S1, S2). The translational fusion PCR product was obtained by PCR amplification with primers (360/361) and pGH254 as template (Supplementary Tables S1, S2). Selection of successful recombinants was mediated by spreading cells on NA plates containing ampicillin, X-gal, and 1μg/ml anhydrotetracycline. The proper integration of transcriptional or translational fusions was confirmed by diagnostic PCR and then sequenced.

### Construction of the Δ*crp* Mutant

Deletion of the *crp* gene was achieved by replacement of the *crp* locus with a kanamycin resistance cassette using λ red-mediated recombination as previously described (Datsenko and Wanner, 2000). The kanamycin resistance cassette was amplified from pKD4 plasmid template with primers (362/363; Supplementary Tables S1, S2) containing a 50bp homologous extension with upstream and downstream of the coding sequence of *crp*. The resulting PCR product was then used to transform, by electroporation, MG1655 strain harboring pKD46 plasmid. Deletion of *crp* was confirmed by diagnostic PCR.

### Genetic Screening for the Identification of Genes Involved in Regulation of *ytfK* Expression

A collection of plasmids containing 6His-tagged genes (minus GFP) from the ASKA library (Kitagawa et al., 2005) was used to transform the TB28 translational fusion (*ytfK* TL P1+P2) reporter strain by electroporation. Cells were diluted and spread on NA plates supplemented with chloramphenicol, 50 or 200μM IPTG and 80μg/ml X-gal. Petri plates were incubated overnight at 37°C and colonies were screened for their dysregulated expression of *ytfK* (Supplementary Figure S1), then streaked and their plasmids were sequenced.

### 6His-CRP Tagged Protein Purification

The pEG25:6His-*crp* plasmid expressing *crp* with an N-terminal 6His tag, under the control of the T5 lac promoter inducible by IPTG was used to transform BL21 (DE3) cells. Several transformants were grown at 37°C overnight in LB medium containing 100μg/ml of ampicillin. Culture was then diluted 50-fold in 2l of the same medium and incubated at 37°C with shaking until OD_600nm_ 0.6. The expression of the 6His-CRP protein was induced by adding IPTG at a final concentration of 0.5mM for 2h. Bacteria were harvested (9,000 × g, 20min at 4°C) and the pellet was stored at – 80°C. Cells were resuspended and incubated in lysis buffer (50mM Tris-HCl pH 8, 300mM NaCl, 1mM EDTA, 10mM imidazole, 0.5mg/ml lysozyme, 1mM phenylmethylsulfonyl fluoride (PMSF), 20μg/ml DNase and 15mM MgCl_2_) for 1.5h at 4°C with gentle shacking and were disrupted using three cycles of French press lysis steps. The cleared lysate was recovered by centrifugation (6,080 × g, 25min) and 6His-CRP protein was purified by ion metal affinity chromatography using a 5ml Nickel (HiTrapHP) column on an AKTA pure 25 (GE healthcare) and desalted using Hiprep 26/10 Desalting column, as previously described (Germain et al., 2019; Supplementary Figure S2A). The last step in 6His-CRP purification was achieved by size-exclusion chromatography (SEC) using a HiLoad 26/600 Superdex 200pg. column pre-equilibrated with 50mM Tris-HCl pH 8, 500mM NaCl, 500mM KCl, 2mM β-mercaptoethanol, and 2% glycerol. The SEC chromatogram is visualized in Supplementary Figure S2B. The purity of the 6His-CRP was verified by SDS-gel electrophoresis (Supplementary Figure S2C). The 6His-CRP protein was stored at −80°C in storage buffer (40mM Tris-HCl pH 8, 238mM NaCl, 22% glycerol, and 1.6mM β-mercaptoethanol).

### Electrophoretic Mobility Shift Assay

5' or 3'-Cy5-labeled DNA fragments of the *ytfK* promoter region were obtained by PCR amplification with appropriate oligonucleotides (Supplementary Table S2). DNA fragments (5nM) were incubated for 15min at 37°C with 6His-CRP (12.5, 25, 65, 130, 195, 300, or 500μM) or not, in 14μl of the binding buffer [10mM Tris-HCl pH 7.5, 50mM NaCl, 5mM MgSO4, 1mM DTT (dithiothreitol), 1mg/ml BSA (bovine serum albumin), 200μM cAMP, 10% glycerol and 12μg/ml poly (dI-dC)]. Ten microliters of the sample were then loaded into a 5% polyacrylamide (37.5/1 [wt/wt] acrylamide-bisacrylamide) gel containing 0.25X TBE (tris-borate EDTA) pH 7.5, 10% glycerol and 200μM cAMP. After migration (10 volts/cm, 1h, 4°C) in migration buffer (TBE 0.25X, 20μM cAMP), the bands were visualized using phosphoImaging (GE Healthcare). The DNA fragment of *ytfK* promoter region deleted for the putative CRP-binding site was obtained by PCR extension of overlapping DNA fragments using appropriate oligonucleotides (Supplementary Table S2).

### β-Galactosidase Activity Assay

Cells expressing transcriptional fusion (*ytfK* P1+P2) were grown at 37°C in MOPS minimal medium containing 0.025% of glucose and 0.4mM KH_2_PO_4_. At the indicated time point, OD_600nm_ was measured and 200μl of the culture was taken at indicated times and incubated with 800μl of Z-Buffer (0.06M Na_2_HPO_4_ 7H_2_O, 0.04M NaH_2_PO_4_ H_2_O, 0.01M KCl, 0.001M MgSO_4_ 7H_2_O and 0.05M β-mercaptoethanol). Twenty microliters of chloroform was added and cells were vortexed 3×10s followed by incubation at room temperature for 2min. Fifty microliters of the sample was incubated with 150μl of Z-Buffer pre-heated to 28°C. ONPG was added at a final concentration of 0.67mg/ml and the β-galactosidase activity was measured according to the Miller method (Miller, 1992) by following the OD_420nm_ with TECAN microplate reader.

### *In vivo* (p)ppGpp Assessment

Bacteria were grown at 37°C overnight in MOPS medium containing 2mM KH_2_PO_4_ and 0.2% glucose. Cells were then diluted 100-fold in 500μl of fresh MOPS medium containing 0.4mM phosphate (KH_2_PO_4_) and 0.025% glucose. Cells were continuously and uniformly labeled with 5μl of ^32^P (0.37MBq/μl, PerkinElmer) and grown at 37°C with shaking (440rpm). Fifty microliters of samples were taken at the indicated times and 20μl of 21M ice-cold formic acid was added to stop the reaction. Samples were kept on ice for 20min and then stored at −20°C. Cell extracts were recovered by centrifugation (14,000 × g for 60min at 4°C). Five microliters of each sample were spotted into PEI Cellulose TLC (thin layer chromatography) plates (purchased from MercK-Millipore) and resolved with 1.5M KH_2_PO_4_ pH 3.4. TLC plates were then revealed by PhosphoImaging (GE Healthcare) and analyzed using ImageQuant software (GE Healthcare). The amount of ppGpp was normalized by the amount of pppGpp, ppGpp and GTP for the wild-type strain and the Δ*ytfK* mutant and by the amount of ppGpp and GTP for the Δ*relA* and Δ*relA*Δ*ytfK* mutants.

### Cell Survival to Prolonged Carbon Starvation

Overnight cultures of M9 minimum medium containing 2% of glucose were diluted 100-fold in fresh M9 medium containing 0.025% of glucose. Bacteria were cultivated during 120h with shaking at 37°C. Evaporated water was measured and compensated by regularly adding the missing volume of water throughout the culture. Aliquots were taken at indicated hours, cells were serially diluted and plated on NA medium. Plates were then incubated at 37°C overnight and the number of CFU/ml was determined.

## Results

### *ytfK* Is Transcribed From Two-Independent Promoters and the cAMP Level Plays a Key Regulatory Role in *ytfK* Expression

**Figure 1:**
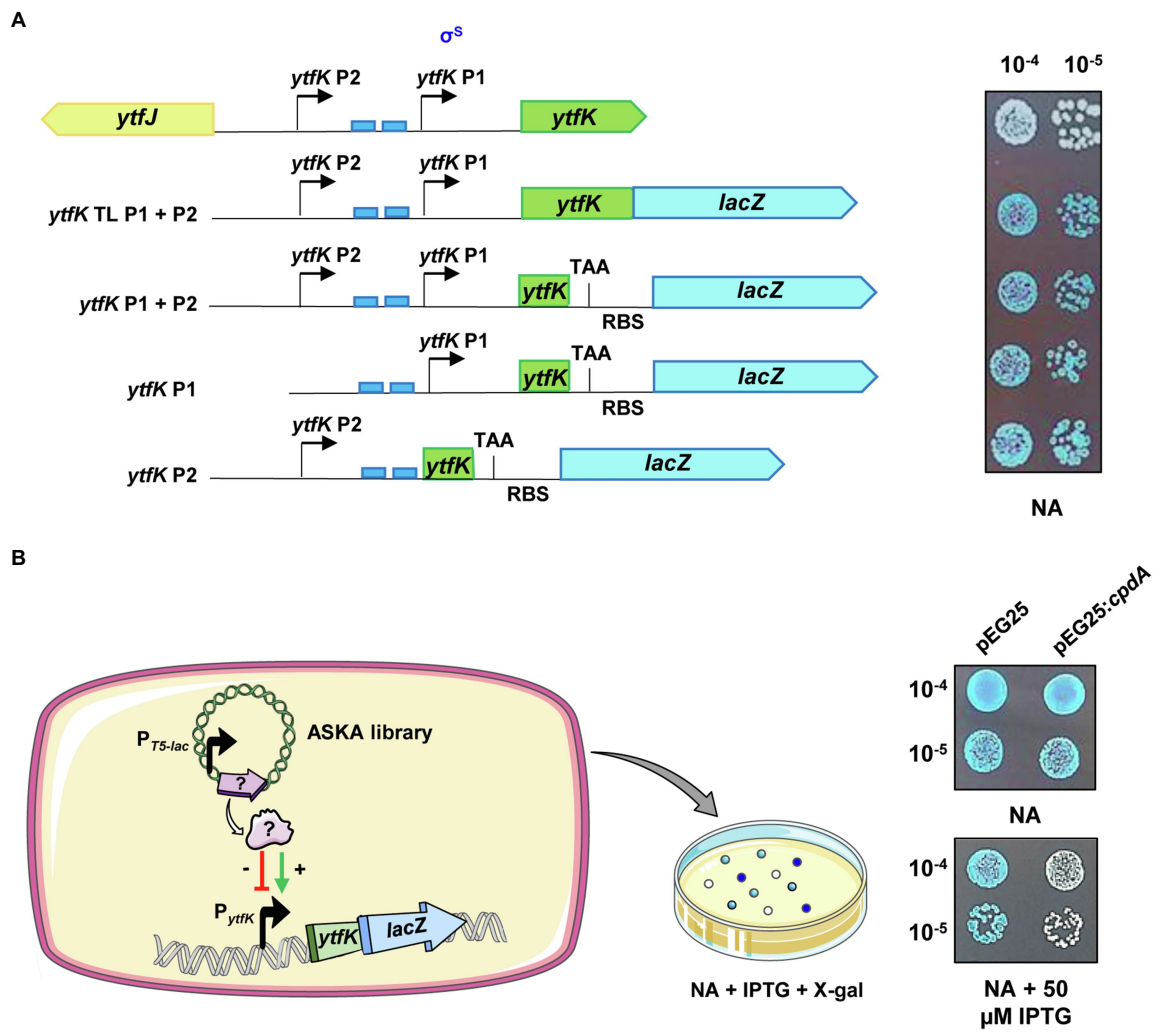
*ytfK* is transcribed from two distinct promoters. **(A)** *ytfK* promoter region. The divergent *ytfK* and *ytfJ* genes are represented by green and yellow arrows, respectively. The previously identified (P1) and the putative (P2) transcriptional start sites are indicated by black arrows. The two *pho* boxes recognized by the response regulator PhoB are visualized by blue rectangles. The following schematic representations describe translational and transcriptional *lacZ* fusions used in this study and on the right the corresponding expression level as judged by colorimetric visualization of *lacZ* activity on X-gal plate. Briefly, stationary-phase cultures of TB28 cells harboring translational (*ytfK* TL P1+P2) or transcriptional (*ytfK* P1+P2, *ytfK* P1, or *ytfK* P2) fusions were serially diluted and 5µl of 10^−4^ and 10^−5^ dilutions were spotted on NA rich solid medium containing X-gal. Results are representative of three independent experiments. **(B)** Schematic representation of the genetic screen based on overexpression of *E. coli* genes from a pooled plasmid of ASKA library (Kitagawa et al., 2005) in TB28 cells harboring translational fusion (*ytfK* TL P1+P2) and screening of candidates having dysregulated level of YtfK as judged by colorimetric visualization of *lacZ* level on X-gal NA plates. On the right, stationary-phase cultures of cells expressing translational fusion (*ytfK* TL P1+P2) and harboring pEG25 or pEG25:*cpdA* plasmids were serially diluted and spotted on NA rich solid medium supplemented or not with 50μM IPTG and X-gal. Results are representative of three independent experiments.

*ytfK* is transcribed as a monocistronic unit (Figure 1A). One promoter, referenced in this study as P1 promoter, has been shown to be recognized by the sigma factor σ^S^ (Lacour and Landini, 2004), which is proposed to be involved in the induction of *ytfK* expression at the onset of stationary phase (Salgado et al., 2013). In addition, two *pho* boxes recognized by PhoB are located upstream the P1 transcriptional start site and are required for induction of *ytfK* expression in response to phosphate starvation (Baek and Lee, 2006; Yoshida et al., 2011; Figure 1A). Moreover, a second putative P2 promoter is predicted (Lacour and Landini, 2004) upstream the P1 promoter but has not yet been experimentally validated.

In order to dissect the promoter region of *ytfK* and to analyze the genetic regulation of *ytfK* expression, we first generated (Figure 1A; see Materials and Methods) chromosomal translational (*ytfK* TL P1+P2) and several truncated transcriptional *lacZ* fusions (*ytfK* P1, *ytfK* P2, or *ytfK* P1+P2) to follow promoter activity on X-gal plates. As shown in Figure 1A, *ytfK* expression is driven from two-independent promoters when cells were spotted on nutrient rich agar, thus confirming the existence of a distal P2 promoter.

To gain further insight on how *ytfK* is regulated, we used cells harboring the translational fusion (*ytfK* TL P1+P2) and searched for activators and inhibitors of *ytfK* expression by overexpressing *E. coli* genes from the ASKA library pool (Kitagawa et al., 2005) and by using screening assay on X-gal plates for selection of clones with dysregulated *lacZ* activity (Figure 1B). The ASKA library encompasses almost all *E. coli* genes cloned into the high-copy-number vector pCA24N, under the control of a P_T5-*lac*_ promoter inducible by IPTG (Kitagawa et al., 2005). Out of the approximately 40,000 clones screened, 29 candidates were selected and their plasmids were sequenced (Table 1; Supplementary Figure S1).

**Table 1:** Genes identified by overexpressing each *E. coli* gene from the ASKA library (Kitagawa et al., 2005) and by screening for the impaired regulation of translational fusion (*ytfK* TL P1+P2).

Clone	Gene overexpressed	Function	Regulation of *ytfK* expression
**Cell wall/membrane/envelope biogenesis**			
A3	*ybaY* ^*^	PF09619 family lipoprotein	+
B4	*mepS* ^*^	peptidoglycan DD-endopeptidase	+
C1	*ydbA* ^*^	putative outer membrane protein N-terminal fragment	+
C2	*mdtP* ^*^	putative multidrug efflux pump outer membrane channel	+
C3	*ydbA* ^*^	putative outer membrane protein N-terminal fragment	+
C4	*mdtP* ^*^	putative multidrug efflux pump outer membrane channel	+
D4	*nanC* ^*^	N-acetylneuraminic acid outer membrane channel	+
D7	*pldA* ^*^	outer membrane phospholipase A	+
D10	*mepS* ^*^	peptidoglycan DD-endopeptidase	+
E2	*ampH* ^*^	peptidoglycan DD-carboxypeptidase	+
E6	*pbpG* ^*^	peptidoglycan DD-endopeptidase	+
**Signal transduction mechanisms**			
B2	*cpdA*	cAMP phosphodiesterase	−
D3	*ydfK*	qin prophage, cold shock protein	−
E11	*ycgZ*	putative two-component system connector protein	−
B12	*dgcJ* ^*^	putative diguanylate cyclase	+
**Cell motility**			
B8	*yraK* ^*^	putative fimbrial adhesin	+
C7	*sfmF* ^*^	putative fimbrial protein	+
C10	*yhcA* ^*^	putative fimbrial chaperone	+
E4	*yfcP* ^*^	putative fimbrial protein	+
E9	*sfmF* ^*^	putative fimbrial protein	+
E12	*fimF* ^*^	type I fimbriae minor subunit	+
**Amino acid transport and metabolism**			
A5	*ldtC* ^*^	L,D-transpeptidase	+
C11	*ldtB* ^*^	L,D-transpeptidase	+
E5	*ycjN* ^*^	putative ABC transporter periplasmic binding protein	+
**Carbohydrate transport and metabolism**			
A6	*yihS*	sulfoquinovose isomerase	−
E8	*glK*	glucokinase	−
**Lipid transport and metabolism**			
A1	*plsX*	putative phosphate acyltransferase	+
**Energy production and conversion**			
F2	*xdhA*	putative xanthine dehydrogenase molybdenum binding subunit	−
**Translation, ribosomal structure and biogenesis**			
A11	*rlmN*	23S rRNA m^2^ A2503 methyltransferase	−

*Genes whose the overexpression upregulates or downregulates the ytfK expression are indicated by (+) or (−), respectively. Clones are designated by their relative position in the Supplementary Figure S1. Genes encoding membrane proteins are marked with an asterisk*.

We confirmed, after re-cloning into a more suitable physiological plasmid harboring a tight IPTG-inducible P_T5-*lac*_ promoter (pEG25), that the ectopic overexpression of *cpdA*, encoding a cAMP phosphodiesterase, strongly decreases *ytfK* expression level (Figure 1B). Therefore, this result suggests that the intracellular level of cAMP plays an important role in adjusting *ytfK* expression level.

### *ytfK* Expression Level Is Induced by the cAMP-CRP Complex at the Transcriptional Level

**Figure 2:**
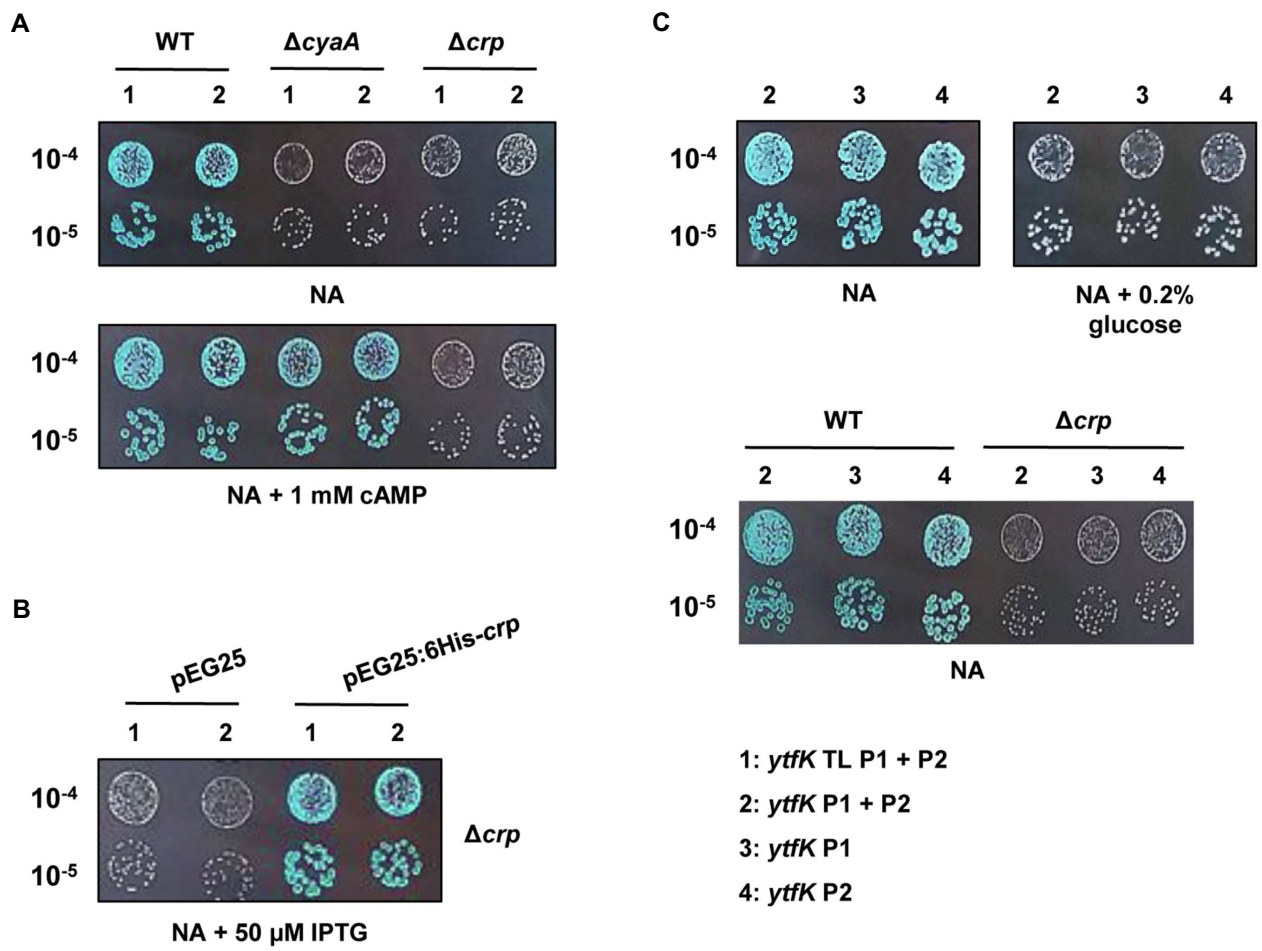
*ytfK* expression is controlled by cAMP level and requires the presence of the master regulator CRP. **(A)** Stationary-phase cultures of WT (TB28), Δ*cyaA* or Δ*crp* cells expressing translational (1: *ytfK* TL P1+P2) or transcriptional (2: *ytfK* P1+ P2) *lacZ* fusions were serially diluted and 5µl of 10^−4^ and 10^−5^ dilutions were spotted on X-gal NA plates supplemented or not with 1mM cAMP. **(B)** Stationary-phase cultures of TB28 Δ*crp* cells harboring pEG25 or pEG25:6His-*crp* plasmids and expressing translational (1: *ytfK* TL P1+P2) or transcriptional (2: *ytfK* P1+ P2) fusions were serially diluted and spotted on X-gal NA plates supplemented with 50μM IPTG. **(C)** Stationary-phase cultures of WT (TB28) or Δ*crp* cells expressing transcriptional (2: *ytfK* P1+ P2; 3: *ytfK*+P1 or 4: *ytfK*+P2) *lacZ* fusions were serially diluted and spotted on X-gal NA plates supplemented or not with 0.2% of glucose. Results are representative of three independent experiments.

In *E. coli*, cAMP is degraded by CpdA (Imamura et al., 1996) and is synthesized by the adenylate cyclase CyaA (Notley-McRobb et al., 1997). To understand how cAMP plays an essential role in controlling the YtfK level, we first followed *lacZ* activity of wild-type (TB28 strain) and Δ*cyaA* cells harboring translational or transcriptional *lacZ* fusions. We first observed that deletion of the adenylate cyclase *cyaA* dramatically reduces *ytfK* expression and this regulation occurs at the transcriptional level (Figure 2A). Moreover, adding 1mM of cAMP in the medium fully restored *ytfK* expression (Figure 2A). These results confirm that cAMP is necessary to induce *ytfK* transcription under standard rich conditions.

The CRP is the best-known cAMP target in *E. coli* (Postma et al., 1993). Once activated by cAMP, the cAMP-CRP complex has a central role in the integration of external signals, such as carbon starvation by regulating, in absence of glucose, the expression of several hundred genes involved in the uptake and catabolism of other carbon sources (Zheng et al., 2004; Shimada et al., 2011). We naturally investigated the involvement of CRP in regulation of *ytfK*. Interestingly and similarly to what was observed in a Δ*cyaA* strain, we found that transcriptional expression of *ytfK* is also strongly impaired in the Δ*crp* mutant (Figure 2A). Importantly, this phenotype is fully trans-complemented by pEG25:6His-*crp* (Figure 2B). However, addition of 1mM of cAMP does not restore expression of *ytfK* in the Δ*crp* mutant (Figure 2A). Therefore, these results show that the cAMP-CRP complex positively regulates transcription of *ytfK*.

Glucose is transported into the cell and phosphorylated to glucose-6-phosphate, by the phosphotransferase system (PTS), which is composed of several proteins (i.e., EI, HPr, and EIIA^Glc^; Postma et al., 1993; Bettenbrock et al., 2007). The phosphorylation state of the PTS is lower when glucose is available in the medium, whereas once glucose is consumed, phosphorylated PTS proteins accumulate. The phosphorylated EIIA^Glc^ protein interacts with CyaA and stimulates its activity, thus increasing the intracellular cAMP concentration (Notley-McRobb et al., 1997). In agreement with these data, we observed that the *ytfK* expression is also highly reduced, when cells are grown in a NA rich medium supplemented with 0.2% of glucose (Figure 2C). In addition, we found that cells expressing transcriptional (*ytfK* P1) or (*ytfK* P2) fusions are both submitted to carbon catabolite repression (Figure 2C), suggesting that in the absence of glucose, the cAMP-CRP complex promotes *ytfK* transcription from both promoters.

Taken together, our results support that the cAMP-CRP complex is required to regulate *ytfK* transcription in response to glucose availability.

### The cAMP-CRP Complex Directly Binds the *ytfK* P2 Promoter to Regulate Its Expression

**Figure 3:**
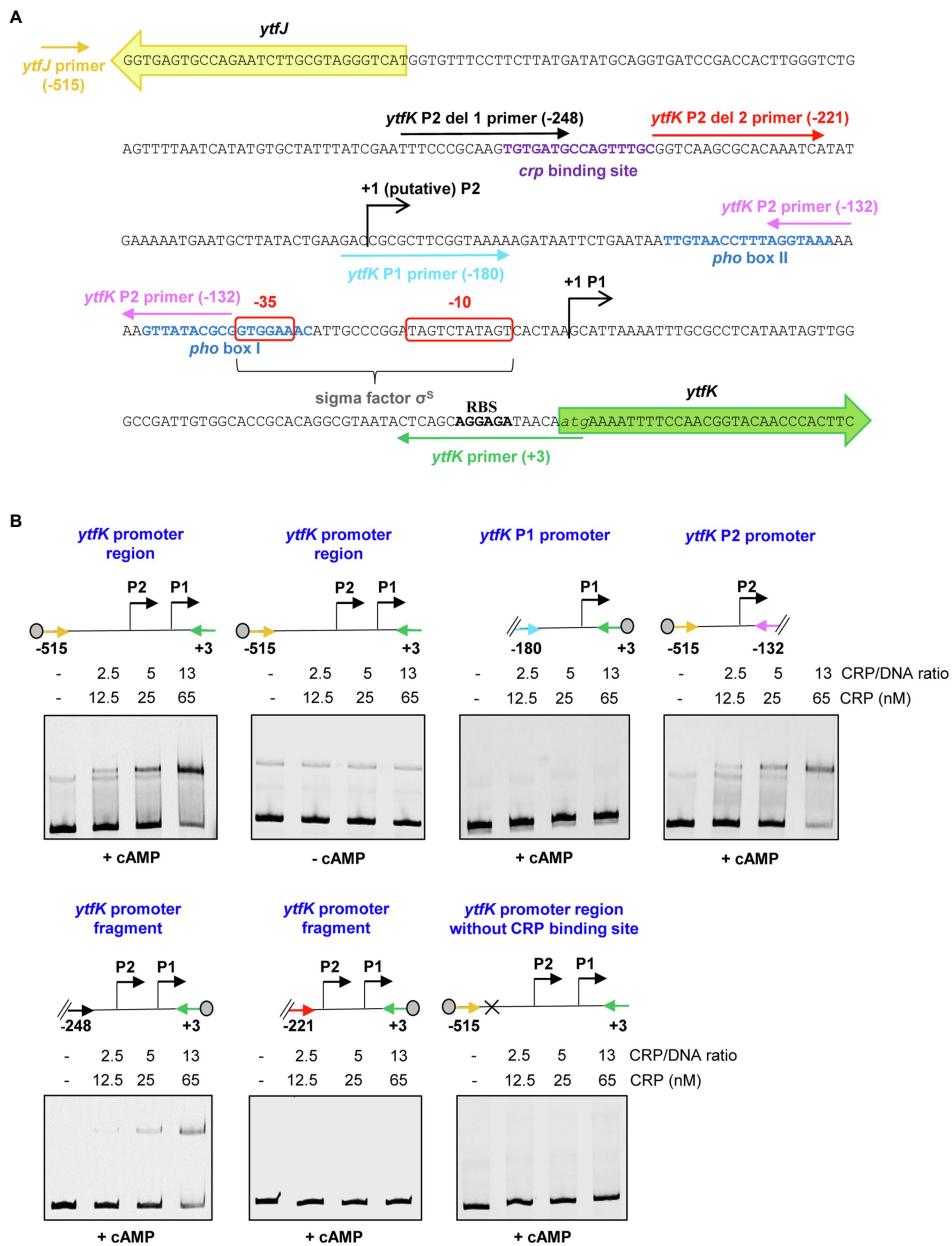
The cAMP-CRP complex forms a tight complex with the *ytfK* P2 promoter. **(A)** Intergenic sequence encompassing the *ytfK* promoter region. The coding sequences of *ytfK* and *ytfJ* are shown by green and yellow arrows, respectively. The −35 and−10 sequences of the P1 promoter are similar to the consensus sequences recognized by the sigma factor σ^S^ (Lacour and Landini, 2004; Salgado et al., 2013) and are boxed as red. The *pho* boxes (I and II) and the putative CRP-binding site are written in blue and purple, respectively. The transcriptional start sites of the P1 and putative P2 promoters are indicated by black arrows. The putative ribosome-binding site (RBS) located upstream of the *ytfK* coding sequence and its start codon is indicated in bold and in italic letters, respectively. Primers used to generate DNA fragments for electrophoretic mobility shift assay are indicated by colored arrows and the relative position to the start codon of *ytfK* is indicated. **(B)** EMSA using the CRP protein (0, 12.5, 25 or 65nM) and the indicated DNA fragments (with relative position to the ATG start codon of *ytfK*). Position of the Cy5 dye is represented by a gray circle. Samples were incubated for 15min at 37°C, separated by native 5% PAGE and bands were visualized using phosphoImaging (Typhoon, GE Healthcare). Results are representative of three independent experiments.

To address whether the cAMP-CRP complex directly regulates *ytfK* expression, we first produced and purified the CRP protein by two consecutive chromatography steps (affinity and size exclusion; Supplementary Figure S2) and electrophoretic mobility shift assay (EMSA) were performed with 5' or 3'-Cy5-labeled DNA fragments selected from the *ytfK* promoter region (Figure 3A).

Using DNA covering the entire promoter sequence, a band shift is observed even in the presence of a large amount of poly (dI-dC) competitor (Figures 3A,B). Moreover, this interaction occurs only in the presence of cAMP (Figures 3A,B), as is commonly observed for *E. coli* CRP homologs (Green et al., 2014). Importantly, no band shift is detected when the intragenic sequence of *hofB is* used as internal negative control as previously shown (Cameron and Redfield, 2006; Supplementary Figure S3). In addition, the CRP binding to the *ytfK* promoter sequence appears upon addition of a 2.5-fold molar excess CRP/DNA and the estimated dissociation constant (K_D_) is around 25nM range suggesting that CRP has a strong affinity for the *ytfK* promoter region (Figures 3A,B). Moreover, no additional shifted band of lower mobility was observed even when the CRP/DNA mixing ratio was raised up to 100 (Supplementary Figure S4) suggesting existence of a single-binding site. Finally, and consistent with this observation, we show that CRP specifically interacts with the P2 promoter region and that no binding was observed with the P1 promoter region (Figures 3A,B).

To further determine the accurate localization of the CRP-binding site, we made several truncated deletions in the *ytfK* P2 promoter region (Figure 3A). Using this approach, we observed that cAMP-CRP complex binds a region located between 248 and 221 base pairs upstream the *ytfK* start codon (Figures 3A,B). We searched in this short region of interest for DNA sequence similar to the known consensus sequence (5'-TGTGAT-N6-TCACA-3') recognized by CRP (Shimada et al., 2011) and we found as a potential CRP-binding site, the 5'-TGTGATGCCAGTTTGC-3' sequence located at 229bp upstream the start codon (Figure 3A). To validate this prediction, the putative CRP-binding site has been deleted and we observed that CRP no longer binds to the *ytfK* promoter region (Figures 3A,B). These results suggest that CRP induces *ytfK* transcription by its binding to the 5'-TGTGATGCCAGTTTGC-3' sequence located in the P2 promoter and that induction of transcription from P1 promoter is likely to be indirect (Figures 3A,B).

### *ytfK* Contributes to SpoT-Mediated (p)ppGpp Accumulation During Glucose Starvation

**Figure 4:**
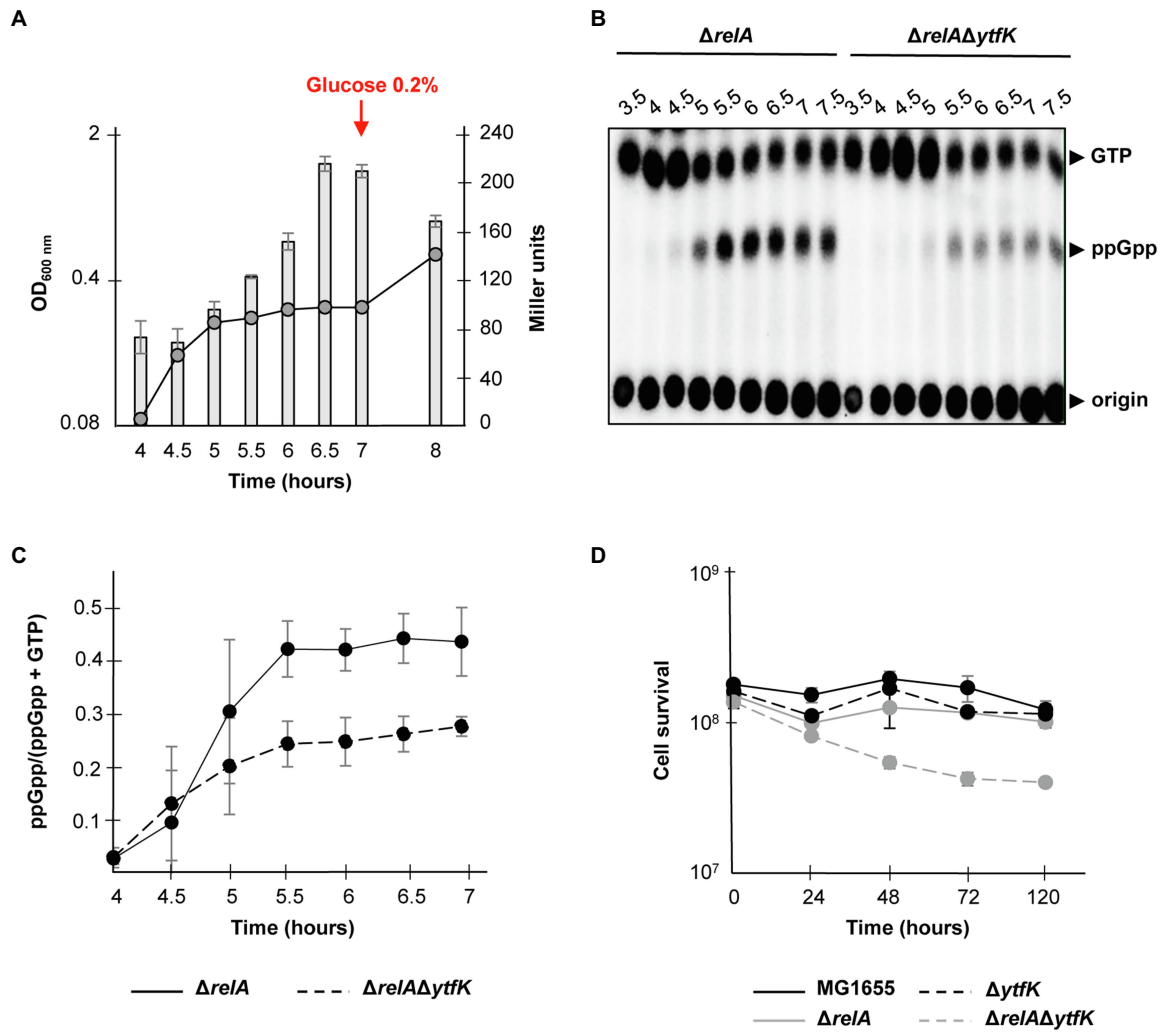
*ytfK* is required for SpoT-dependent (p)ppGpp accumulation during glucose exhaustion. **(A)** Growth curve and β-galactosidase activity assay of WT cells in glucose limited minimal medium. WT (TB28) cells harboring the transcriptional fusion (*ytfK* P1+ P2) were grown in MOPS minimum medium containing 0.025% glucose. The OD_600nm_ values of the cultures (curve) were taken and β-galactosidase activity (bar chart) was measured at indicated times. After 7h of growth, glucose was added at a final concentration of 0.2%. Error bars indicate the standard deviations of averages of three independent experiments. **(B)** *In vivo* (p)ppGpp accumulation in response to glucose exhaustion. The Δ*relA* and Δ*relA*Δ*ytfK* mutants were labeled with ^32^P and grown at 37°C in low-phosphate MOPS medium containing 0.025% of glucose (see Materials and Methods). Samples were taken at indicated times prior to nucleotides extraction and are separated by TLC. Representative autoradiograph of the TLC plates is shown and quantification is provided in **(C)**. Error bars indicate the standard deviations of averages of four independent experiments. **(D)** YtfK is required for long-term survival during carbon starvation in absence of *relA*. Cells of MG1655 (WT; black line) and isogenic deletion strains Δ*relA* (gray line), Δ*ytfK* (black dotted line) and the Δ*relA*Δ*ytfK* double mutant (gray dotted line) were grown in M9 minimum medium containing limited concentration of glucose (0.025%). Cell survival (log scale) was determined at indicated time. Error bars indicate the standard deviations of averages of three independent experiments.

Under carbon starvation, SpoT promotes the (p)ppGpp accumulation (Xiao et al., 1991; Gentry and Cashel, 1996; Murray and Bremer, 1996), but little is known about how this environmental change is sensed by bacteria and linked to SpoT-dependent (p)ppGpp accumulation. As mentioned before, YtfK protein level plays an important role in adjusting intracellular (p)ppGpp level in *E. coli* (Germain et al., 2019). Given that cAMP-CRP complex plays an important role in carbon sources catabolism and positively regulates the *ytfK* expression, we naturally investigated the role of YtfK in SpoT-dependent (p)ppGpp accumulation under glucose starvation. For that purpose, we first followed expression of *ytfK* during glucose exhaustion, a condition known to trigger SpoT-dependent (p)ppGpp accumulation (Xiao et al., 1991; Gentry and Cashel, 1996). As shown in Figure 4A and consistent with the observed positive regulatory role of cAMP-CRP complex, *ytfK* expression gradually increased 30min after growth arrest due to glucose exhaustion. We then compared (p)ppGpp accumulation in the wild-type strain and the Δ*ytfK* mutant in response to glucose exhaustion. However, and as shown in Supplementary Figure S5, both strains seem to have similar kinetics of (p)ppGpp accumulation in response to glucose exhaustion. Importantly, in the absence of amino acids, both RelA and SpoT contribute to (p)ppGpp accumulation during carbon source or diauxic growth transition (Gentry and Cashel, 1996; Fernández-Coll and Cashel, 2018). Therefore, we further decided to address the role of YtfK in response to glucose starvation in cells devoid of RelA. We observed that compared to the wild-type strain, deletion of *relA* causes earlier growth arrest and a 30min delayed kinetic of (p)ppGpp accumulation (Figures 4B,C and Supplementary Figures S5, S6). Interestingly, we observed that compared to the Δ*relA* mutant, the Δ*relA*Δ*ytfK* mutant exhibits a strong decrease in ppGpp accumulation in response to glucose exhaustion (Figures 4B,C). Hence, YtfK contributes to the full SpoT-dependent (p)ppGpp accumulation in response to carbon starvation.

SpoT-dependent (p)ppGpp accumulation is also known to be essential for survival during prolonged glucose starvation (Nystöm, 1994). Therefore, we addressed the role of YtfK in cell survival during carbon starvation. While survival of the wild-type, Δ*relA* and Δ*ytfK* strains are not significantly affected after 5days of carbon starvation (Figure 4D), the Δ*relA*Δ*ytfK* double mutant rapidly loses its viability after 24h and retains less than 30% of viability after 5days of prolonged carbon starvation (Figure 4D). Taken together, our results show that regulation of *ytfK* is a fine-tuned regulated process that allows cells to rapidly adapt and survive during long-term carbon starvation by triggering SpoT-dependent (p)ppGpp accumulation.

### ytfK Coordinates Growth Resumption During Glucose-Lactose Diauxic Shift

**Figure 5:**
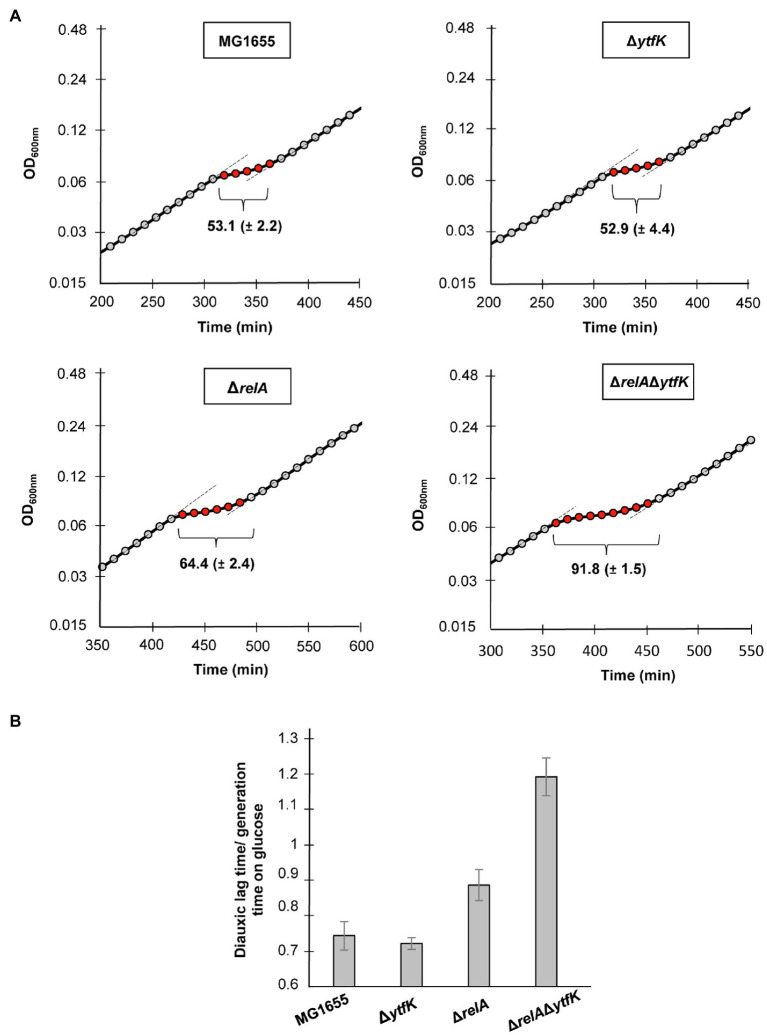
YtfK contributes to diauxic shift adaptation. **(A)** Cells of MG1655 (WT) and isogenic deletion strains Δ*relA*, Δ*ytfK* and the Δ*relA*Δ*ytfK* were grown in MOPS minimum medium containing 0.025% of glucose and 0.4% of lactose. Growth was monitored every 10min using Tecan microplate reader. Representative growth is provided. The length of diauxic lag times (visualized by brackets) is calculated from three independent experiments and was normalized to the generation time during growth on glucose **(B)**. Error bars indicate the standard deviations of averages of three independent experiments.

When *E. coli* is cultivated in a medium containing various carbon sources, the glucose is preferentially consumed until its exhaustion. (p)ppGpp accumulation rapidly occurs resulting in a transitional growth arrest allowing the establishment of regulatory networks that coordinate the resumption of growth on another carbon source (Harshman and Yamazaki, 1971; Traxler et al., 2006). This phenomenon causes biphasic growth, well known as diauxie (Jacob and Monod, 1961). Importantly a tight control of (p)ppGpp level governs the length of the diauxic lag (Fernández-Coll and Cashel, 2018). We therefore addressed the role of YtfK in diauxic shift. For this purpose, we cultivated the wild-type strain, the simple mutants Δ*relA*, Δ*ytfK* and the double-mutant Δ*relA*Δ*ytfK* in MOPS minimal medium containing a limiting concentration of glucose (0.025%) and an excess of lactose (0.4%). The diauxic lag times were calculated and normalized by generation times on glucose, as previously described (Fernández-Coll and Cashel (2018). We showed that the wild-type strain and the Δ*ytfK* mutant display a similar diauxic lag times of 53min (Figure 5A) and a diauxic lag time/generation time on glucose close to 0.7 (Figure 5B). Moreover and as previously reported (Fernández-Coll and Cashel, 2018), we observed that compared to the wild-type strain, deletion of *relA*, significantly increases the diauxic lag time (64min; Figure 5A) and the ratio diauxic lag time/generation time on glucose (0.9; Figure 5B). Interestingly, the Δ*relA*Δ*ytfK* double mutant displays an important extended diauxic lag time (92min; Figure 5A) and the ratio diauxic lag time/generation time on glucose reaches 1.2 (Figure 5B), showing that YtfK also plays an important role in SpoT-dependent (p)ppGpp accumulation during diauxie.

Taken together, these results showed that YtfK plays a key role in sensing carbon starvation though the cAMP-CRP complex and transducing the signal to SpoT to orchestrate (p)ppGpp accumulation and thus coordinate cellular metabolism.

## Discussion

In *E. coli*, two homologous enzymes work in concert to control (p)ppGpp level: the (p)ppGpp synthetase RelA and the bifunctional synthetase/hydrolase SpoT. While RelA possesses only (p)ppGpp synthetic activity responding primarily to amino acid starvation (or other stresses that would ultimately cause amino acid starvation), SpoT has both hydrolytic and synthetic activities and functions as a central protein responding to an extreme variety of stress (Xiao et al., 1991; Seyfzadeh et al., 1993; Spira et al., 1995; Vinella et al., 2005). Tight regulation of the synthetic and hydrolytic intracellular activities is crucial for rapidly adjusting (p)ppGpp level and several molecular mechanisms have been reported (Wout et al., 2004; Battesti and Bouveret, 2006; Jiang et al., 2007; Lee et al., 2018; Germain et al., 2019). We previously observed that ectopic production of YtfK is sufficient to trigger SpoT-dependent (p)ppGpp accumulation in absence of external stress and that the YtfK/SpoT ratio controls the intracellular amount of (p)ppGpp (Germain et al., 2019). Therefore, the aim of this work was to further decipher the genetic control involved in the regulation of *ytfK* expression and its impact on the cell physiology. We first confirmed experimentally that the *ytfK* promoter region comprises two promoters (Figure 1A), as previously expected (Lacour and Landini, 2004; Salgado et al., 2013). We then systematically searched for trans-regulatory element of *ytfK* expression using screening assay. This approach leads us to the observation that cAMP level is critical for *ytfK* expression (Figures 1B, 2A and Supplementary Figure S1). In addition, we showed that *ytfK* is submitted to carbon catabolite repression and that in the absence of glucose, the cAMP-CRP complex promotes *ytfK* transcription from both promoters (Figures 2A,C). Further analysis highlights that CRP directly binds the 5'-TGTGATGCCAGTTTGC-3' sequence located in the P2 promoter. Moreover CRP probably regulates *ytfK* expression *via* the P1 promoter by an indirect unknown mechanism (Figures 3A,B).

**Figure 6:**
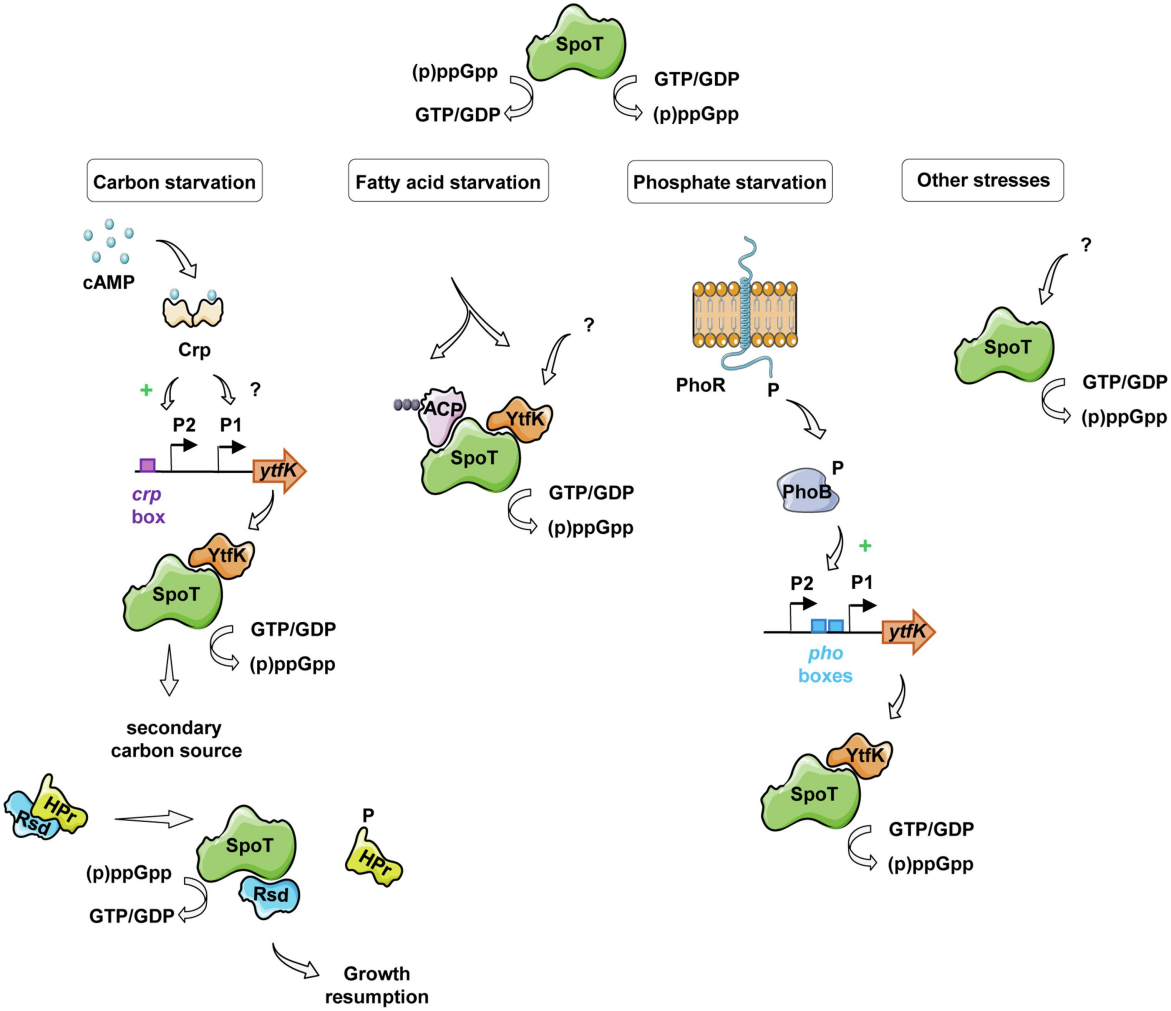
Schematic view of SpoT integration signals and its regulation in response to various starvations in *E. coli*. Under glucose starvation, an increase of cAMP concentration activates the CRP protein, which induces *ytfK* transcription by direct binding to the *ytfK* P2 promoter and by an unknown and probably indirect mechanism for the *ytfK* P1 promoter. YtfK thus interacts with the catalytic domains of SpoT (Germain et al., 2019), promoting the accumulation of (p)ppGpp. The availability of a secondary carbon source releases the anti-σ^70^ Rsd protein from HPr, which can interact with the C-terminal regulatory domain of SpoT, rebalancing its (p)ppGpp synthesis activity toward hydrolysis allowing growth resumption (Lee et al., 2018). In response to fatty acid starvation, YtfK also promotes SpoT-dependent accumulation of (p)ppGpp (Germain et al., 2019) and on the other hand, the nature of the fatty acid intermediates bound to ACP differs and induces conformational changes in the protein leading to SpoT-dependent (p)ppGpp accumulation (Battesti and Bouveret, 2006). During phosphate starvation, the PhoR-PhoB two-component system mediates signal transduction that results to induce *ytfK* transcription which could orchestrate SpoT-dependent (p)ppGpp accumulation (Spira et al., 1995; Spira and Yagil, 1998; Baek and Lee, 2006; Yoshida et al., 2011; Iwadate and Kato, 2017).

Interestingly, earlier studies have observed an interplay between the (p)ppGpp and the cAMP-CRP regulon in response to carbon starvation, where (p)ppGpp is at the apex of the signaling pathway and maximizes induction of CRP-activated genes (Traxler et al., 2006). Importantly, while regulatory mechanisms orchestrating carbon catabolite repression in *E. coli* have been intensively characterized, signaling pathway regulating SpoT-dependent (p)ppGpp accumulation in response to carbon availability remains poorly understood. Here, we partially elucidated this mechanism by showing that the YtfK contributes to SpoT-dependent (p)ppGpp accumulation (Figures 4B,C) and cell viability (Figure 4D) in response to glucose starvation. We also observed that a residual (p)ppGpp accumulation persists in the *ΔrelAΔytfK* double mutant during carbon starvation. Upon fatty acid starvation, the acyl carrier protein (ACP) interacts with SpoT to promote the accumulation of (p)ppGpp (Battesti and Bouveret, 2006; Figure 6). Importantly, ACP and fatty acid metabolism could also be a relay for responding to carbon source starvation and be responsible for the residual SpoT activity observed in the *ΔrelAΔytfK* cells. Indeed, carbon exhaustion would lead to fatty acid starvation through shrinkage of the acetyl-CoA pool produced during glycolysis. It is interesting to note that YtfK and ACP interact with two different regions of SpoT. YtfK binds the catalytic domains in the N-terminal region (Germain et al., 2019), while ACP binds the TGS domain in C-terminal regulatory region (Battesti and Bouveret, 2006; Figure 6). It is therefore tempting to speculate that binding of the both proteins may work in concert to maximize (p)ppGpp level in response to glucose starvation. It seems to be a striking opposite parallel between the mechanisms of SpoT regulation upon fatty acid starvation *via* ACP and the SpoT regulation by the anti-σ^70^ Rsd during carbon downshift. Indeed, Rsd binding to the TGS domain of SpoT promotes hydrolase activity upon carbon downshift and plays a physiological role in controlling cell growth recovery during diauxic shift (Figure 6). Our observation that YtfK contributes to the SpoT-dependent (p)ppGpp accumulation during diauxie and seems also to be important for growth resumption during glucose-lactose diauxic shift (Figure 5) is consistent with the notion that a tightly coordinated balance between synthesis versus hydrolysis activity rather than the absolute (p)ppGpp level is important for faster adaption during diauxic shift.

In addition to the regulation presented in this study, *ytfK* transcription is subjected to complex regulatory network. Indeed, *ytfK* also contains, upstream of its coding sequence, two *pho* boxes similar to the consensus sequence recognized by the response regulator PhoB and transcriptome analyses showed that *ytfK* is induced 15-fold by PhoB during phosphate starvation (Baek and Lee, 2006; Yoshida et al., 2011). Moreover, the PhoR-PhoB two-component system has been shown to play key role in triggering SpoT-dependent (p)ppGpp accumulation during phosphate starvation (Spira et al., 1995; Spira and Yagil, 1998: Figure 6). Importantly, deletion of *ytfK* also affects cell viability and (p)ppGpp accumulation under phosphate starvation (Iwadate and Kato, 2017; Germain et al., 2019). Interestingly, *ytfK* is also induced and involved in H_2_O_2_ tolerance (Iwadate and Kato, 2017). In addition, we previously described that *ytfK* is also induced during fatty acid starvation causing accumulation of (p)ppGpp and cell survival (Figure 6; Germain et al., 2019). However, the exact nature of this regulation remains to be determine. Finally, *ytfK* expression is also under the control of the ferric uptake regulator Fur (Zhang et al., 2005), involved in response to iron limitation (Stojiljkovic et al., 1994; Hantke, 2001, 2002; McHugh et al., 2003), another signal known to trigger SpoT-dependent (p)ppGpp accumulation *in vivo* (Vinella et al., 2005). Importantly, the amount of SpoT protein has been estimated around hundreds of molecules per cell (Pedersen and Kjeldgaard, 1977) and to our knowledge, no transcriptional regulation of *spoT* has been directly linked to the environmental control of SpoT activities. Therefore, the regulation of *ytfK* appears as an emerging key node in the SpoT-mediated activation of stringent response in *E. coli* (Figure 6).

Finally, aside from identification of the role of cAMP in the regulation of *ytfK*, our genetic screening assay enables the identification of many proteins, probably acting indirectly, involved in a variety of key functions, such as cell wall biogenesis, cell motility, amino acid metabolism, carbohydrates and lipids transport, energy maintenance and signal transduction (Table 1).

Therefore, any perturbation in these metabolic pathways may influence YtfK protein level and therefore could impact SpoT activities and modulate intracellular (p)ppGpp level. For instance, it is well known that the overexpression of membrane proteins disrupts membrane integrity by limiting the capacity of proteins translocation into the membrane and by increasing the aggregation of cytoplasmic proteins due to the titration of chaperones, which leads to broad perturbations of the proteome and is responsible for inefficient ATP synthesis (Wagner et al., 2007). Overexpression of membrane proteins highly induces *ytfK* expression (Table 1 and Supplementary Figure S1), suggesting that YtfK protein may play a role in response to cell envelope disruption which could impact SpoT activities and modulate the intracellular level (p)ppGpp.

Therefore, beyond a better understanding of the genetic regulation, our systematic analysis of the trans-regulatory elements can indirectly allow us to better understand the metabolic pathways behind the regulation of SpoT activities and can lead to the identification of new stressful conditions in which SpoT and its partners intervene (Figure 6).

## Data Availability Statement

The raw data supporting the conclusions of this article will be made available by the authors, without undue reservation.

## Author Contributions

EM, LM, and EG designed the study and discussed the results. LM performed the experiments. EM and LM wrote the manuscript. EM acquired the funding. All authors contributed to the article and approved the submitted version.

## Funding

This work was supported by the European Research Council starting grant (ERC StG) under the European Union’s Horizon 2020 and innovation program grant agreement no. 714934 “Stringency” to EM.

## Conflict of Interest

The authors declare that the research was conducted in the absence of any commercial or financial relationships that could be construed as a potential conflict of interest.

## Publisher’s Note

All claims expressed in this article are solely those of the authors and do not necessarily represent those of their affiliated organizations, or those of the publisher, the editors and the reviewers. Any product that may be evaluated in this article, or claim that may be made by its manufacturer, is not guaranteed or endorsed by the publisher.
