# Supplementary material for: Regulation of ytfK by cAMP-CRP Contributes to SpoT-Dependent Accumulation of (p)ppGpp in Response to Carbon Starvation YtfK Responds to Glucose Exhaustion
Source: Front Microbiol. 2021 Nov 4;12:775164. doi: 10.3389/fmicb.2021.775164 (PMC8600398; doi:10.3389/fmicb.2021.775164)
Supplement: Supplementary file 1 [file Data_Sheet_1.pdf]

Supplementary Material to regulation of *ytfK* by cAMP-CRP contributes to SpoT-dependent accumulation of (p)ppGpp in response to carbon starvation.

Laura Meyer, Elsa Germain and Etienne Maisonneuve*

Laboratoire de Chimie Bactérienne, Institut de Microbiologie de la Méditerranée,

CNRS-Aix Marseille Univ (UMR7283), Marseille, France

*Correspondence: emaisonneuve@imm.cnrs.fr

Running Title: YtfK responds to glucose exhaustion.

Keywords: (p)ppGpp, Stringent Response, *ytfK*, cAMP, CRP, glucose starvation, *E. coli*

Supplementary Figure legends

Supplementary Figure S1: Candidates whose overexpression impaired *ytfK* regulation.

Out of 40 000 clones from the genetic screen using the ASKA library (Kitagawa et al., 2005), 65 potential candidates were grown in LB medium and spotted on NA solid medium supplemented with 50 or 200µM of IPTG. Translational fusion (*ytfK* TL P1+P2) reporter strain harboring pCA24N:*cpdA* are indicated by a red square and as a control, cells harboring the empty vector pCA24N are visualized by a black square.

Supplementary Figure S2: Purification of the 6His:CRP protein.

(**A**) SDS-PAGE of samples collected during 6His:CRP purification using nickel column. M: molecular weight markers, T: total fraction, CL: cleared lysate, FT: flow through, W: column washing, E1-E6: eluted fractions. After migration, the 12% acrylamide gel was stained with coomassie blue. (**B**) Size-exclusion chromatography (SEC). The elution volume (from a HiLoad 16/600 Superdex 200 column) is plotted on the horizontal axis and the 280nm absorbance on the vertical axis. SEC elution fraction highlighted by black arrow was analyzed by 12% SDS-gel electrophoresis (**C**). Molecular weight markers are expressed in kDa.

Supplementary Figure S3: CRP is not able to bind the *hopF* promoter.

Electrophoretic mobility shift assay was performed (see Materials and Methods) with the *hopF* promoter region obtained by PCR amplification with indicated primers (**Table S2**) and the 6His:CRP protein (at indicated concentrations) in presence of cAMP (200µM). Results are representative of three independent experiments.

Supplementary Figure S4: CRP binds the *ytfK* promoter region at a single binding site.

Electrophoretic mobility shift assay was performed (see Materials and Methods) with the entire *ytfK* promoter region and the 6His:CRP protein (at indicated concentrations) in presence of cAMP (200µM). Results are representative of three independent experiments.

Supplementary Figure S5: The wild-type strain and the Δ*ytfK* mutant accumulate similar intracellular level of (p)ppGpp under carbon starvation.

(**A**) The wild-type strain and the Δ*ytfK* mutant were grown at 37°C in low-phosphate MOPS minimum medium containing 0.025% of glucose. The OD_{600nm} values of the cultures were taken every 30 min. (**B**) For analysis of (p)ppGpp level by TLC under glucose exhaustion, cells were labeled with P^{32} and samples were taken at indicated times. The nucleotides were extracted as described in Materials and Methods and revealed by PhosphoImaging (**B**) and quantified (**C**). Error bars indicate the standard deviations of averages of three independent experiments.

Supplementary Figure S6: The Δ*relA* and Δ*relA*Δ*ytfK* mutants exhibit similar growth arrest under glucose exhaustion.

The Δ*relA* and Δ*relA*Δ*ytfK* mutants were grown at 37°C in low-phosphate MOPS minimum medium containing 0.025% of glucose. The OD_{600nm} values of the cultures were taken every 30 min. Error bars indicate the standard deviations of averages of three independent experiments.

Supplementary References

Baba, T., Ara, T., Hasegawa, M., Takai, Y., Okumura, Y., Baba, M., et al. (2006). Construction of *Escherichia coli* K-12 in-frame, single-gene knockout mutants: the Keio collection. *Mol. Syst. Biol.* 2, 2006.0008. doi:10.1038/msb4100050.

Blank, K., Hensel, M., and Gerlach, R. G. (2011). Rapid and highly efficient method for scarless mutagenesis within the *Salmonella enterica* chromosome. *PLoS One* 6, e15763. doi:10.1371/journal.pone.0015763.

Datsenko, K. A., and Wanner, B. L. (2000). One-step inactivation of chromosomal genes in *Escherichia coli* K-12 using PCR products. *Proc. Natl. Acad. Sci. U. S. A.* 97, 6640–5. doi:10.1073/pnas.120163297.

Germain, E., Guiraud, P., Byrne, D., Douzi, B., Djendli, M., and Maisonneuve, E. (2019). YtfK activates the stringent response by triggering the alarmone synthetase SpoT in *Escherichia coli*. *Nat. Commun.* 10, 5763. doi:10.1038/s41467-019-13764-4.

Kitagawa, M., Ara, T., Arifuzzaman, M., Ioka-Nakamichi, T., Inamoto, E., Toyonaga, H., et al. (2005). Complete set of ORF clones of *Escherichia coli* ASKA library (a complete set of *E. coli* K-12 ORF archive): unique resources for biological research. *DNA Res.* 12, 291–9. doi:10.1093/dnares/dsi012.

Supplementary Table S1: Bacterial strains and plasmids used in this study.

Strains	Genotype or other relevant characteristics	Source or reference
MG1655	K-12 F^{-} λ^{-} *ilvG rfb-50 rph-1*	Laboratory stock
DH5α	*supE44* Δ*lacU* (Φ80 *lacZ*ΔM15) *hsdR17 recA1 endA1 gyrA96 thi-1 relA1*	Laboratory stock
TB28	MG1655 Δ*lacIZYA* K-12	Laboratory stock
BL21 (DE3)	F^{-} *ompT gal*	New England Biolabs
Δ*relA*	MG1655 Δ*relA*::*FRT*	(Germain et al., 2019)
Δ*ytfK*	MG1655 Δ*ytfK*::*FRT*	(Germain et al., 2019)
Δ*relA*Δ*ytfK*	MG1655 Δ*relA*::*FRT* Δ*ytfK*::*FRT*	(Germain et al., 2019)
ytfK TL P1 + P2	TB28 *ytfK*Ω$P_{ytfK\ P1\ +\ P2}$:*ytfK-lacZ*	This work
ytfK P1 + P2	TB28 Δ*ytfK*Ω$P_{ytfK\ P1\ +\ P2}$:*lacZ*	This work
ytfK P1	TB28 Δ*ytfK*Ω$P_{ytfK\ P1}$:*lacZ*	This work
ytfK P2	TB28 Δ*ytfK*Ω$P_{ytfK\ P2}$:*lacZ*	This work
Δ*crp*	MG1655 Δ*crp*::*FRT*::*kan*::*FRT*	This work
Δ*cyaA*	K-12 Δ*cyaA*:*FRT*::*kan*::*FRT*	Keio collection (Baba et al., 2006)
ytfK TL P1 + P2 Δ*crp*	TB28 *ytfK*Ω$P_{ytfK\ TL\ P1\ +\ P2}$:*ytfK-lacZ* Δ*crp*::*FRT*	This work (P1 transduction from Δ*crp* in *ytfK* TL P1 + P2 strain)
ytfK P1 + P2 Δ*crp*	TB28 Δ*ytfK*Ω$P_{ytfK\ P1\ +\ P2}$:*lacZ* Δ*crp*::*FRT*	This work (P1 transduction from Δ*crp* in *ytfK* P1 + P2 strain)
ytfK P1 Δ*crp*	TB28 Δ*ytfK*Ω$P_{ytfK\ P1}$:*lacZ* Δ*crp*::*FRT*	This work (P1 transduction from Δ*crp* in *ytfK* P1 strain)
ytfK P2 Δ*crp*	TB28 Δ*ytfK*Ω$P_{ytfK\ P2}$:*lacZ* Δ*crp*::*FRT*	This work (P1 transduction from Δ*crp* in *ytfK* P2 strain)
ytfK TL P1 + P2 Δ*cyaA*	TB28 *ytfK*Ω$P_{ytfK\ TL\ P1\ +\ P2}$:*ytfK-lacZ* Δ*cyaA*::*FRT*	This work (P1 transduction from Δ*cyaA* in *ytfK* TL P1 + P2 strain)
ytfK P1 + P2 Δ*cyaA*	TB28 Δ*ytfK*Ω$P_{ytfK\ P1\ +\ P2}$:*lacZ* Δ*cyaA*::*FRT*	This work (P1 transduction from Δ*cyaA* in *ytfK* P1 + P2 strain)

Plasmid	Genotype or other relevant characteristics	Source or reference
pEG25	pUC *bla* $P_{T5\text{-}lac}$ promoter, Amp^R	(Germain et al., 2019)
pKD4	Source of the kanamycin resistance cassette for ʎ red recombinaison, Amp^R	(Datsenko and Wanner, 2000)
pKD46	Helper plasmid for expression of ʎ red recombinase, Amp^R	(Datsenko and Wanner, 2000)
pCP20	Plasmid for expression of FLP recombinase, Amp^R	(Datsenko and Wanner, 2000)
pWRG99	pKD46 with I-*sceI* under P_{tetA} inducible promoter, Amp^R	(Blank et al., 2011)
pWRG100	Source of the chloramphenicol resistance cassette with a I-*sceI* recognition site, Cat^R	(Blank et al., 2011)
pGH254	Mini-R1, *lacZYA* transcriptional fusion vector, Kan^R	Laboratory stock
pEG25:6His-*crp*	pEG25; $P_{T5\text{-}lac}$:6His-*crp*	This work (primers 474/475)
pEG25:*cpdA*	pEG25; $P_{T5\text{-}lac}$:*cpdA*	This work (primers 599/605)
pGH254:$P_{ytfK\ P1+P2}$:*lacZ*	pGH254; $P_{ytfK\ P1+P2}$:*lacZ*	This work (primers 98/99)
pGH254:$P_{ytfK\ P1}$:*lacZ*	pGH254; $P_{ytfK\ P1}$:*lacZ*	This work (primers 99/101)
pGH254:$P_{ytfK\ P2}$:*lacZ*	pGH254; $P_{ytfK\ P2}$:*lacZ*	This work (primers 98/100)

Supplementary Table S2: Primers used in this study

Primers	Sequence (from 5' to 3')	Use
474	CCCC**GAATTC**GTCGACTCAAGGAGGTTTTATAAATGCATCACCATCACCATCACGTGCTTGGCAAACCGCAAAC (**EcoRI**)	Clonage of 6His-*crp* in pEG25
475	CCCC**GGATCC**ACGAGTGCCGTAAACGA (**BamH1**)	Clonage of 6His-*crp* in pEG25
599	CCCC**GGATCC**GTCGACTCAAGGAGGTTTTATAATTGGAAAGCCTGTTAACCCT (**BamH1**)	Clonage of *cpdA* in pEG25
605	CCCC**AAGCTT**TCAGTAGCCTTCTGAAGCGG (**HindIII**)	Clonage of *cpdA* in pEG25
98	CCCC**CACGTG**GAATCTTGCGTAGGGTCATGG (**PmlI**)	Clonage of transcriptional fusions (*ytfK* P1 + P2 and *ytfK* P2) in pGH254
99	CCCC**GGATCC**GTATAACCGTCCACGGAACAG (**BamH1**)	Clonage of transcriptional fusions (*ytfK* P1 + P2 and *ytfK* P1) in pGH254
100	CCCC**GGATCC**CGCGTATAACTTTTTTTACCT (**BamH1**)	Clonage of transcriptional fusion (*ytfK* P2) in pGH254
101	CCCC**CACGTG**GAAGACCGCGCTTCGGTAAAA (**PmlI**)	Clonage of transcriptional fusion (*ytfK* P1) in pGH254
353	TTGGGCCGATTGTGGCACCGCACAGGCGTAATACTCAGCAGGAGATAACACGCCTTACGCCCCGCCCTGC	Chromosomal integration of I-*SceI*::*cat* cassette (*ytfK* P1 + P2 and *ytfK* P2 fusions)
354	CAGGAGCCGTCTTATTACTGCATAGCACTTTTTACTGCATAGCACTTTGGCTAGACTATATTACCCTGTT	Chromosomal integration of I-*SceI*::*cat* cassette (*ytfK* P1 + P2, *ytfK* P1 and *ytfK* P2 fusions)
355	GAATCTTGCGTAGGGTCATGGTGTTTCCTTCTTATGATATGCAGGTGATC	Chromosomal integration of transcriptional fusions (*ytfK* P1 + P2 and *ytfK* P2)
356	CAGGAGCCGTCTTATTACTGCATAGCACTTTTTACTGCATAGCACTTTGGTTATTTTTGACACCAGACCAA	Chromosomal integration of transcriptional fusions (*ytfK* P1 + P2, *ytfK* P1 and *ytfK* P2)

357	TGATGTCCGAAGTTAACCGTCAGGTTATGC GTCTGCAAACAGAGATGGCTCGCCTTACGC CCCGCCCTGC	Chromosomal integration of I-*SceI*::*cat* cassette (*ytfK* TL P1 + P2 fusion)
358	CAGGAGCCGTCTTATTACTGCATAGCACTTT TTACTGCATAGCACTTTGGCTAGACTATATT ACCCTGTT	Chromosomal integration of I-*SceI*::*cat* cassette (*ytfK* TL P1 + P2 fusion)
360	GTCAGGTTATGCGTCTGCAAACAGAGATGG CTGCAGCGGCCGGCGGAGGGACCATGATTA CGGATTCACT	Chromosomal integration of *lacZ* gene (*ytfK* TL P1 + P2 fusion)
361	CAGGAGCCGTCTTATTACTGCATAGCACTTT TTACTGCATAGCACTTTGGTTATTTTTGACA CCAGACCAA	Chromosomal integration of *lacZ* gene (*ytfK* TL P1 + P2 fusion)
577	CATCGGCAACAGCAGGCAGGTGAGTGCCAG AATCTTGCGTAGGGTCATGGCGCCTTACGC CCCGCCCTGC	Chromosomal integration of I-*SceI*::*cat* cassette (*ytfK* P1 fusion)
708	AACAGCAGGCAGGTGAGTGCCAGAATCTTG GAAGACCGCGCTTCGGTAAA	Chromosomal integration of transcriptional fusions (*ytfK* P1)
362	AGCGGCGTTATCTGGCTCTGGAGAAAGCTT ATAACAGAGGATAACCGCGCGTGTAGGCTG GAGCTGCTTC	Deletion of *crp* gene
363	CGGGGGAAACAAAATGGCGCGCTACCAGG TAACGCGCCACTCCGACGGGATCCTCCTTA GTTCCTATTCC	Deletion of *crp* gene
ytfK P2 del 1 primer (-248)	TTTCCCGCAAGTGTGATGCC	EMSA
ytfK P2 del 2 primer (-221)	GGTCAAGCGCACAAATCATA	EMSA
ytfK P2 primer (-132)	CCCCGGATCCCGCGTATAACTTTTTTTACCT	EMSA
ytfK P1 primer (-180)	CCCCCACGTGGAAGACCGCGCTTCGGTAAA A	EMSA
619	GCCTACCGCATCCGCTT	EMSA
621	CCAGGTTTCCCAGCACTTTTAAT	EMSA

ytfJ primer (-515)	AGTACTCGCACTTTTCCCAC	EMSA and deletion of TGTGATGCCAGTTTGC site in *ytfK* promoter
ytfK primer (+3)	CATTGTTATCTCCTGCTGAG	EMSA and deletion of TGTGATGCCAGTTTGC site in *ytfK* promoter
633	TTTGTGCGCTTGACCCTTGCGGGAAATTCG	Deletion of TGTGATGCCAGTTTGC site in *ytfK* promoter
635	CGAATTTCCCGCAAGGGTCAAGCGCACAAA	Deletion of TGTGATGCCAGTTTGC site in *ytfK* promoter

Restriction sites are indicated in bold letters.
