# Supplementary material for: Regulation of ytfK by cAMP-CRP Contributes to SpoT-Dependent Accumulation of (p)ppGpp in Response to Carbon Starvation YtfK Responds to Glucose Exhaustion
Source: Front Microbiol. 2021 Nov 4;12:775164. doi: 10.3389/fmicb.2021.775164 (PMC8600398; doi:10.3389/fmicb.2021.775164)
Supplement: Supplementary file 2 [file Data_Sheet_2.PDF]

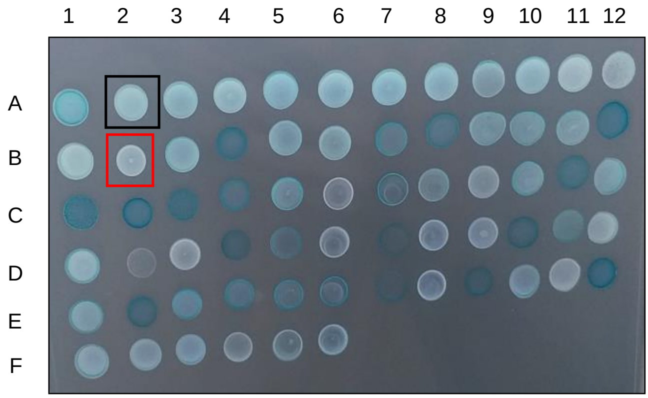


**50µM IPTG**

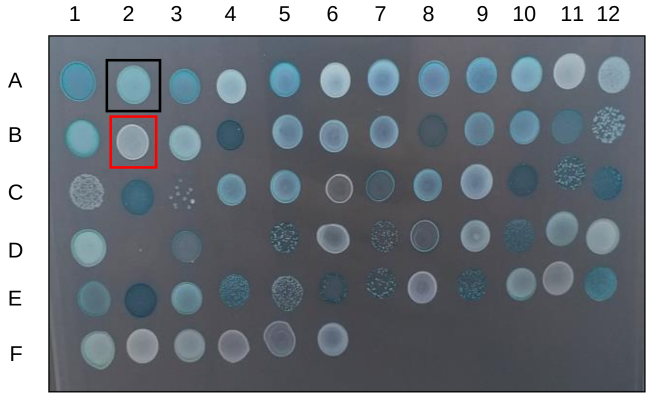


**200µM IPTG**

# Supplementary Figure S1:

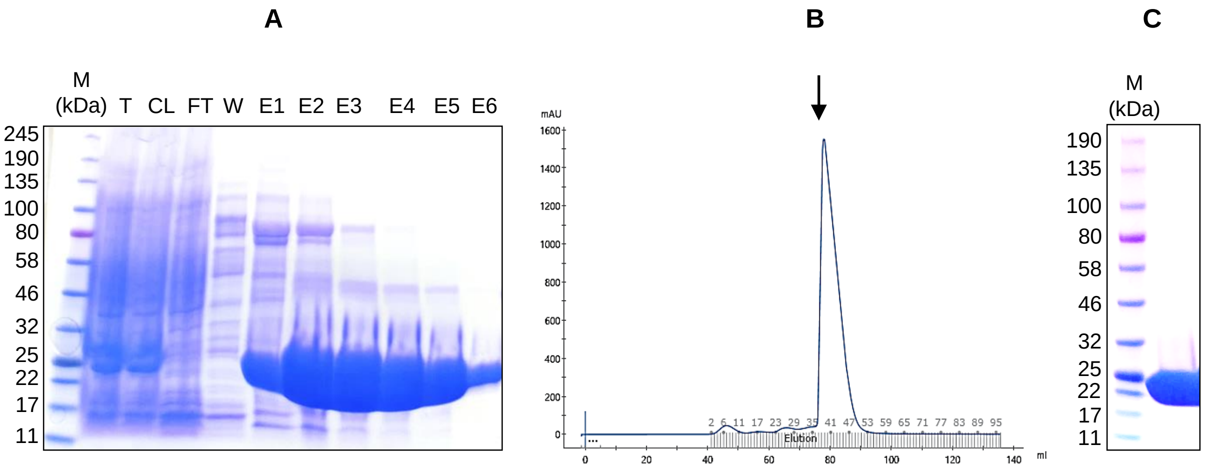


**Supplementary Figure S2:**

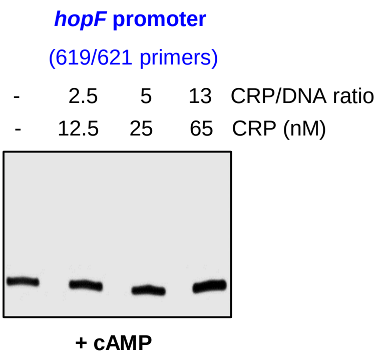


**Supplementary Figure S3:**

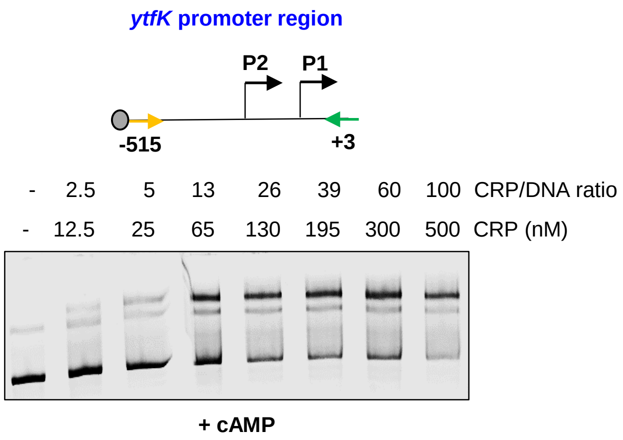


## Supplementary Figure S4:

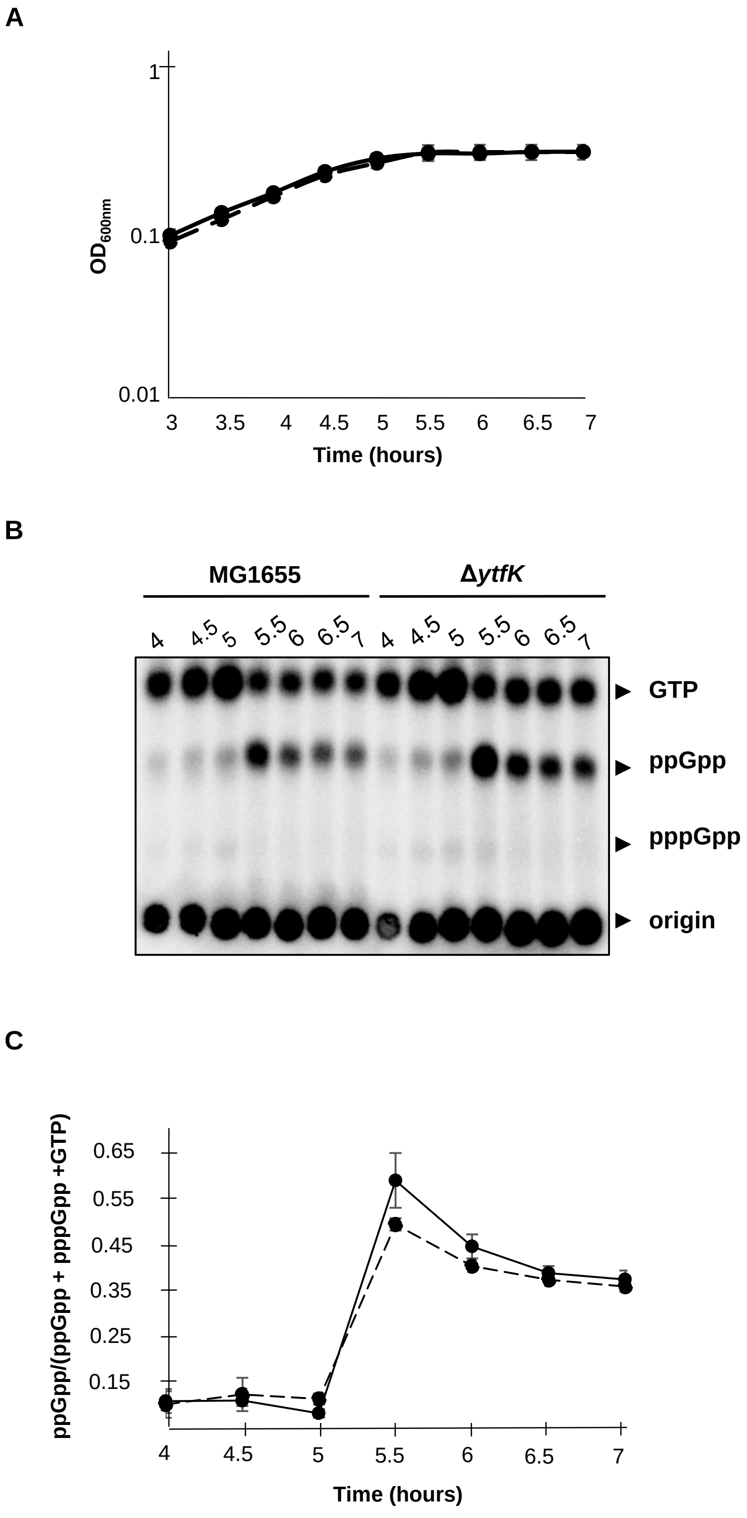


**Supplementary Figure S5:**

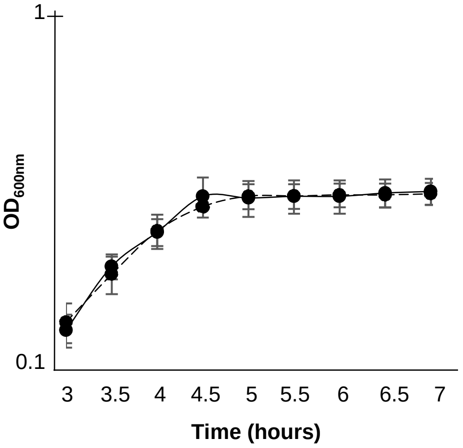


**Supplementary Figure S6:**
